# Aberrantly Expressed tRNA-Val Fragments Can Distinguish Canine Hepatocellular Carcinoma from Canine Hepatocellular Adenoma

**DOI:** 10.3390/genes15081024

**Published:** 2024-08-04

**Authors:** Saki Hashimoto, MD Nazmul Hasan, Mohammad Arif, Nobuhiro Nozaki, Al Asmaul Husna, Yu Furusawa, Takeshi Sogawa, Kaori Takahashi, Tomohide Kuramoto, Aki Noguchi, Masashi Takahashi, Osamu Yamato, Md Mahfuzur Rahman, Naoki Miura

**Affiliations:** 1 Joint Graduate School of Veterinary Medicine, Kagoshima University, 1-21-24 Korimoto, Kagoshima 890-0065, Japan; 2 Joint Faculty of Veterinary Medicine, Kagoshima University, 1-21-24 Korimoto, Kagoshima 890-0065, Japan

**Keywords:** tRNA-Val, canine hepatocellular carcinoma, canine hepatocellular adenoma

## Abstract

Hepatocellular adenoma (HCA) and hepatocellular carcinoma (HCC) can be difficult to differentiate but must be diagnosed correctly as treatment and prognosis for these tumors differ markedly. Relevant diagnostic biomarkers are thus needed, and those identified in dogs may have utility in human medicine because of the similarities between human and canine HCA and HCC. A tRNA-derived fragment (tRF), tRNA-Val, is a promising potential biomarker for canine mammary gland tumors but has not previously been investigated in hepatic tumors. Accordingly, we aimed to elucidate the potential utility of tRNA-Val as a biomarker for canine HCA and HCC using clinical samples (tumor tissue and plasma extracellular vesicles [EVs]) and tumor cell lines with qRT-PCR assays. We also investigated relevant functions and signaling pathways with bioinformatic analyses (Gene Ontology and Kyoto Encyclopedia of Genes and Genomes). tRNA-Val was markedly downregulated in HCC tumor tissue versus HCA tumor tissue and normal liver tissue, and a similar trend was shown in plasma EVs and HCC cell lines versus healthy controls. Based on areas under the receiver operating characteristic curves (AUCs), tRNA-Val significantly distinguished HCC (AUC = 1.00, *p* = 0.001) from healthy controls in plasma EVs and HCC from HCA (AUC = 0.950, *p* = 0.01). Bioinformatics analysis revealed that tRNA-Val may be primarily involved in DNA repair, mRNA processing, and splicing and may be linked to the N-glycan and ubiquitin-mediated proteasome pathways. This is the first report on the expression of tRNA-Val in canine HCC and HCA and its possible functions and signaling pathways. We suggest that tRNA-Val could be a promising novel biomarker to distinguish canine HCC from HCA. This study provides evidence for a greater understanding of the role played by tRNA-Val in the development of canine HCC.

## 1. Introduction

Hepatocellular adenomas (HCAs) are uncommon benign liver tumors that arise from the proliferation of hepatocytes and may transform into hepatocellular carcinoma (HCC), a common and malignant form of liver cancer in humans and dogs [1]. HCCs are the most common hepatic tumors in dogs, accounting for 50–70% of the total, and also represent the sixth most widespread cancer in humans globally [2,3,4,5]. Differentiating between HCA and HCC is a complex task, and as their prognosis and treatments vary, a molecular identification of the tumor type is vital for the patient [6,7]. Accordingly, further research on molecular diagnostics to facilitate the differentiation between these two tumors would be potentially beneficial for oncologists.

Transfer RNA (tRNA)-derived fragments (tRFs) are recently discovered noncoding RNAs (ncRNAs) generated through precise cleavage of pre-mature and mature tRNA molecules [8]. tRFs may contain seed sequences that can target mRNA, resulting in gene silencing. They enter argonaute (AGO) complexes in a similar way to miRNAs [9,10]. tRFs are crucial in various cellular processes, such as cell proliferation, invasion, migration, and drug resistance. These molecules also regulate RNA stability and significantly impact target gene expression [11,12]. tRFs have diagnostic utility in identifying human HCC, ovarian, gastric, and breast cancer [13,14,15,16,17,18,19,20]. A novel 3′ tRNA-derived tRF-Val expression positively correlates with tumor size and depth of tumor invasion in human gastric cancer (GC) tissues [15]. This tRNA-Val has provided new potential therapeutic options for GC. Another study found that aberrant tRF-Val-TAC expression is associated with higher lymph node metastasis in GC patients [21]. In human breast cancer, the downregulation of 5′-tiRNA^Val^ positively correlated with stage progression and lymph node metastasis and could be a potential diagnostic biomarker for breast cancer [22]. In human colorectal cancer, tRNA-Val^AAC/CAC^ is downregulated in stage IV in comparison with stage III and has the potential of using this tRF as a biomarker for cancer diagnosis [23]. Research has also focused on tRFs in different canine cancers, including oral melanoma and MGTs [24,25]. The role of tRFs has not previously been investigated in canine HCC or HCA.

Extracellular vesicles (EVs) are small membrane-bound structures that cells release. These structures are crucial for intercellular communication as they transport vital molecules like DNA, RNA, and proteins between cells [26]. EV-associated tRFs are aberrantly expressed in gastric carcinoma, hypopharyngeal carcinoma, and canine oral melanoma [27,28,29,30]. However, to date, there is a total paucity of research on EV-associated tRFs in canine and human HCC and HCA.

Studying cancer in dogs can improve our understanding of its development in humans. There are marked similarities in biology and appearance between canine and human cancers, which underlines the need for cross-species research that could enhance therapeutic options for both species [31,32]. Canine HCC has many clinical and histological similarities to human HCC, suggesting that treatments for canine HCC could also be utilized for human HCC [2]. Investigating the involvement of tRFs in dogs with HCC offers a valuable opportunity to uncover important information about the development of HCC in humans.

In this study, we determined the expression levels of tRNA-Val in dogs with HCC and dogs with HCA using qRT-PCR analysis. We also validated the expression level of tRNA-Val in clinical canine HCC and HCA tissues, plasma EVs, and HCC cell lines for the first time. Our findings provide useful insight into the diagnosis of canine HCC and HCA.

## 2. Materials and Methods

### 2.1. Clinical Samples

We targeted 28 dogs (15 males and 13 females) aged between 8 and 14 years undergoing surgery at Kagoshima University Veterinary Teaching Hospital or an affiliated clinic in or around Kagoshima City, Japan, as potential tissue/blood donors for the study. Based on liver tissue samples, thirteen of these cases were diagnosed as HCC and fifteen as HCA histopathologically by the hospital’s certified pathologists (Diplomates of the Japanese College of Veterinary Pathologists). These 28 HCC or HCA samples were evaluated in this study. Normal liver tissue samples were obtained from healthy adult beagle dogs (n = 9), purpose-bred for research, at the time of their scheduled necropsy as control samples.

Blood samples were collected from a subset of the tissue donors (n = 20), consisting of healthy controls (n = 6) and dogs with HCA (n = 5) or HCC (n = 9). The patients’ (HCC and HCA donors’) information is presented in Table S1.

The tissue samples were placed in RNAlater immediately after collection. Blood samples were centrifuged after collection to obtain plasma as the supernatant, which was then transferred to new tubes. All samples were stored at −80 °C until downstream analysis. This study was approved by the Ethics Committee of Kagoshima University (KVH210001). All procedures were in accordance with Kagoshima University laws and regulations. All owners gave informed consent for samples from their dogs to be evaluated in this study. Tissue sample collection procedures were carried out by qualified veterinary professionals using the standard clinical procedure.

### 2.2. Cell Lines and Cell Culture

We used two canine HCC cell lines in this study: a fast-proliferating HCC cell line, 95-1044, and an intermediate-proliferating HCC cell line, AZACH [33,34]. Cell lines were cultured using D-MEM medium (Sigma-Aldrich, St. Louis, MO, USA), fetal bovine serum (Thermo Fisher Scientific, Waltham, MA, USA), L-glutamine (Sigma-Aldrich), and spectinomycin (Sigma-Aldrich). All cells were cultured in a humidified incubator with 5% CO_2_ at 37 °C. Cells were counted using an automated cell counter instrument (Luna cell counter II, Logos Biosystems Inc., Annandale, VA, USA).

### 2.3. Isolation of EVs

EVs were isolated from plasma using the Total Exosome RNA and Protein Isolation Kit (Invitrogen, Thermo Fisher Scientific) in accordance with the manufacturer’s protocol. Briefly, an equal volume of phosphate-buffered saline (PBS) was added to a 300 μL aliquot of each plasma sample. The exosome precipitation reagent was added, the test sample was then vortexed well and centrifuged, and the supernatant was discarded. The pellet was reconstituted in 200 μL 1× PBS and kept at −80 °C until analysis.

### 2.4. RNA Extraction

To extract total RNA from tissues and cells, a mirVanaTM RNA Isolation Kit (Thermo Fisher Scientific, Waltham, MA, USA) was used. Total RNA was isolated from EVs using the mirVana PARIS Kit (Thermo Fisher Scientific). To normalize variations, each plasma EV sample was mixed with 5 μL of synthetic cel-miR-39 prior to extraction. To elute total RNA, an elution solution was used. The total RNA concentration was determined using a NanoDrop 2000c spectrophotometer (Thermo Fisher Scientific). An Agilent 2100 Bioanalyzer (Agilent Technologies, Santa Clara, CA, USA) was used to assess RNA quality and integrity. Cells and tissues had RNA Integrity Numbers (RINs) ranging from 8.5 to 9.5.

### 2.5. qRT-PCR Analysis for tRNA-Val

tRNA-Val was selected based on a previously reported NGS dataset (SRA: PRJNA716131) for canine mammary gland tumors [25]. The qRT-PCR protocol has been described previously [24,35,36]. Briefly, the TaqMan MicroRNA Reverse Transcription Kit (Thermo Fisher Scientific) was used with a T100 thermal cycler to reverse transcribe 2 ng (for tissues and cell lines) or 1.25 μL (for plasma EVs) of total RNA into cDNA in accordance with the manufacturer’s instructions. Synthetic cel-miR-39 was spiked into the plasma samples to normalize RNA isolation. The Quant Studio 3 real-time PCR system (Thermo Fisher Scientific) and TaqMan First Advanced Master Mix Kit were used for qRT-PCR. All experiments were carried out twice. The 2^−ΔΔCT^ method was used to assess relative tRNA-Val expression. The internal controls were RNU6B and miR-186 for tissues and EVs, respectively [30]. miR-186 showed more stability as an endogenous control in comparison with RNU6B for plasma EVs using the delta CT comparison method. Therefore, miR-186 was selected as an internal control for EVs. The following custom-made Taqman primer sequences have been designed specifically for qRT-PCR: 5′-GTTTCCGTAGTGTAGTGGTTATCACGTTCGCCTGCC-3′ tRNA-Val (URS000044730B_9606).

### 2.6. Gene Ontology (GO) and Kyoto Encyclopedia of Genes and Genomes (KEGG) Enrichment and Protein-Protein Interaction (PPI) Analysis

The tRNA-Val sequence was submitted to the miRDB database (https://mirdb.org/custom.html, accessed on 22 December 2023) for a custom gene prediction. Next, the targeted genes were submitted to the DAVID bioinformatics database (https://david.ncifcrf.gov/, accessed on 22 December 2023) for GO and KEGG enrichment analyses. The results of the GO and KEGG enrichment analyses were also verified using SRPlot bioinformatics (https://www.bioinformatics.com.cn/en, accessed on 5 January 2024). A string database (https://string-db.org/, accessed on 2 February 2024) was used to identify the PPI interactions among the genes. A confidence score >0.400 was set for the prediction.

### 2.7. Statistical Analysis

Statistical analysis and graph visualization were performed using GraphPad Prism 9 (https://www.graphpad.com/, accessed on 2 October 2023). Normal distribution tests were performed (Anderson–Darling, Shapiro–Wilk, and Kolmogorov–Smirnov) in clinical tissue, plasma EVs, and HCC cell lines. In tissues, plasma EVs, and HCC cell lines, none of these did not pass the normality test. Therefore, the qRT-PCR data were evaluated using a Mann–Whitney U test and one-way analysis of variance (ANOVA) with the Kruskal-Wallis test. The Wilson/Brown method was used to calculate receiver operating characteristic (ROC) curves and areas under the curve (AUCs). *p*-values <0.05 were regarded as statistically significant.

## 3. Results

### 3.1. Expression of tRNA-Val Using qRT-PCR

#### 3.1.1. Relative Expression in Clinical Tissue Samples

**Figure 1:**
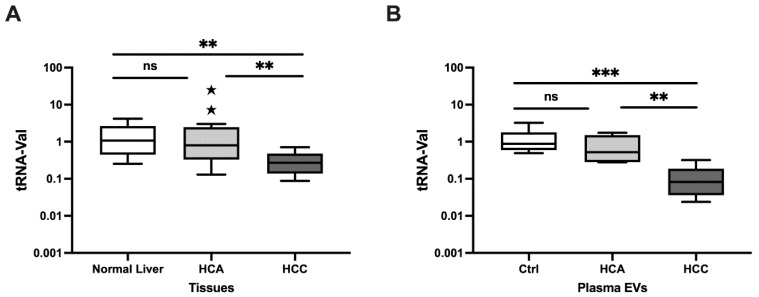
Relative expression in HCA and HCC tissue samples and plasma EVs using qRT-PCR. (**A**). Relative expression levels of tRNA-Val in HCA (n = 15) and HCC (n = 13) versus normal liver (n = 9) tissue. (**B**). Relative expression levels of tRNA-Val HCA (n = 5) and HCC (n = 9) versus healthy control (n = 6) plasma EVs. The *Y*-axis represents relative noncoding RNA expression levels in log10 units. Data were analyzed with one-way ANOVA (nonparametric), followed by the Kruskal-Wallis and Mann–Whitney U tests. *p* <  0.05 (** *p* <  0.01, *** *p* <  0.001) was considered significant. HCA: hepatocellular adenoma, HCC: Hepatocellular carcinoma, ns; not significant, Ctrl: control, EVs: extracellular vesicles.

The relative expression of tRNA-Val was determined for HCA and HCC tissue samples. tRNA-Val expression was significantly downregulated in HCC versus both normal liver (fold change [FC] = 0.22, *p* = 0.003) and HCA (FC = 0.10, *p* = 0.004), but did not significantly differ between HCA and normal liver (Figure 1A). Overall, tRNA-Val in liver tissue significantly differentiated HCC from HCA and normal liver.

#### 3.1.2. Relative Expression in Plasma EVs

Relative tRNA-Val expression was further investigated in plasma EVs, and found to be significantly downregulated in HCC cases versus healthy controls (FC = 0.10, *p* = 0.0002) and HCA cases (FC = 0.19, *p* = 0.008); however, it did not significantly differ between HCA cases and healthy controls (Figure 1B). Overall, tRNA-Val in plasma EVs could distinguish HCC from HCA and healthy controls.

#### 3.1.3. Relative Expression in Canine HCC Cell Lines

**Figure 2:**
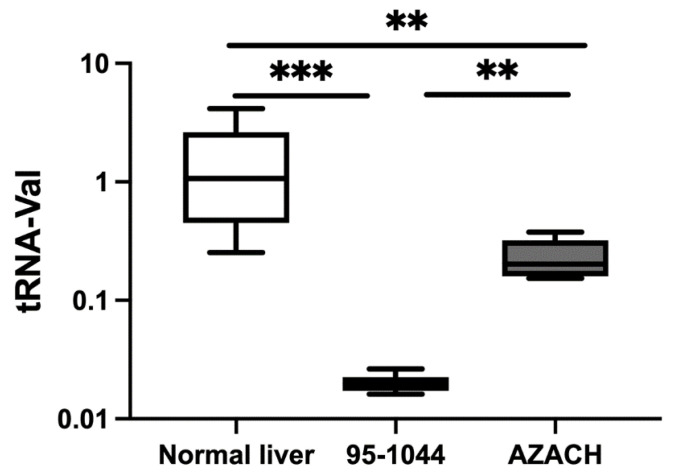
Relative expression in HCC cell lines using qRT-PCR. Relative expression levels of tRNA-Val in HCC-1044 (n = 6) and AZACH (n = 6) versus normal liver tissue (n = 9). The *Y*-axis represents relative noncoding RNA expression levels in log10 units. Data were analyzed with a one-way ANOVA (nonparametric), followed by the Kruskal–Wallis and Mann–Whitney tests. The differences were considered significant when the *p*-value was <0.05 (** *p* < 0.01, *** *p* < 0.001).

We also investigated tRNA-Val expression in two canine HCC cell lines, the fast proliferating 95-1044 and the intermediate-proliferating AZACH cell lines. We found that tRNA-Val expression was decreased significantly in 95-1044 (FC = 0.03, *p* = 0.0002) and AZACH (FC = 0.15, *p* = 0.004) cells versus normal liver tissue, and in 95-1044 cells versus AZACH cells (FC = 0.08, *p* = 0.004) (Figure 2). Overall, these findings were in line with those for clinical samples and show that tRNA-Val is able to distinguish between fast- and intermediate-proliferating cell lines.

### 3.2. Diagnostic Value of tRNA-Val

**Figure 3:**
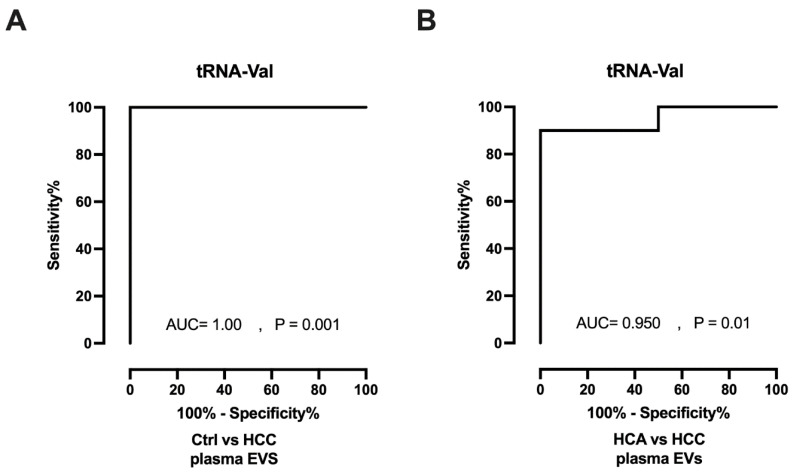
Potential of tRNA-Val as a diagnostic biomarker. ROC curves for tRNA-Val as a differentiator between HCC (n = 9) and Ctrl (n = 6), (**A**), and HCA (n = 5), (**B**). Ctrl: control; HCA: hepatocellular adenoma; HCC: hepatocellular carcinoma; EVs: extracellular vesicles.

ROC curves were plotted, and AUCs were calculated to determine the diagnostic efficacy of tRNA-Val. In plasma EVs, tRNA-Val showed strong diagnostic efficacy for distinguishing HCC from healthy controls (AUC = 1.00, *p* = 0.001; Figure 3A) and from HCA (AUC = 0.950, *p* = 0.01; Figure 3B). However, tRNA-Val was unable to distinguish between HCA and healthy controls (AUC = 0.708, *p* = 0.286; Supplementary Figure S1). In summary, tRNA-Val could serve as a diagnostic biomarker to differentiate HCC from both HCA and healthy controls.

### 3.3. Predicted Targets for tRNA-Val and Enrichment Analyses

**Figure 4:**
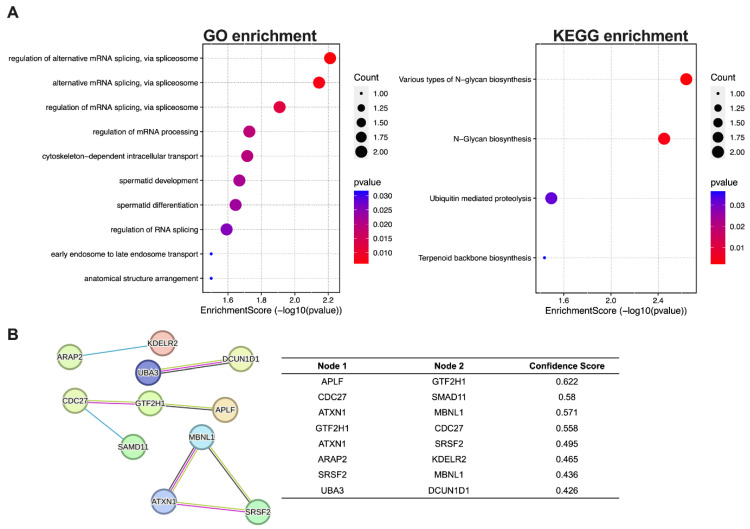
Enrichment and protein–protein interaction (PPI) analysis of target genes of tRNA-Val. (**A**) GO and KEGG enrichment analyses and (**B**) PPI of tRNA-Val. Results were considered significant when *p* < 0.05. The lower-panel table shows the interaction confidence score between nodes 1 and 2. GO: Gene Ontology; KEGG: Kyoto Encyclopedia of Genes and Genomes.

tRFs are known to target genes by binding to AGO complexes, based on complementarity to their seed sequence, in a fashion resembling the seed-sequence-mediated targeting by microRNAs [9]. tRNA-Val target genes were predicted using the miRDB custom gene prediction program. GO enrichment and KEGG enrichment analyses were performed using the targeted gene list to identify the functional roles and pathways in which tRNA-Val is involved. GO enrichment analysis revealed a potential association between tRNA-Val and regulation of mRNA processing and splicing (Figure 4A, Tables S2 and S3). KEGG enrichment analysis revealed the potential involvement of tRNA-Val in various types of N-glycan biosynthesis and ubiquitin-mediated proteolysis. To further elucidate the intrinsic mechanism, a PPI network was established based on a gene-to-gene confidence score using the STRING database (https://string-db.org/, accessed on 2 February 2024) (Figure 4B). Target genes involved in the PPI network are summarized in Table S4. The tRNA-Val target genes included those such as APLF, which is primarily involved in DNA repair; GTF2H1, which regulates transcription by RNA polymerase II; CDC 27, which acts on cell cycle division; and SMAD11, which acts as a negative regulator of transcription by RNA polymerase II; ATX1, which involves transcription regulation and binding; MBNL1, which regulates alternative splicing and RNA binding; SRSF2, a critical splicing factor that regulates RNA splicing; and UBA3, which regulates protein degradation.

## 4. Discussion

We aimed to elucidate the diagnostic efficacy of tRNA-Val as a tRF biomarker for distinguishing canine HCA from canine HCC by evaluating its expression in liver tissue, plasma EVs, and HCC cell lines. To the best of our knowledge, this is the first report on tRF expression in canine liver tumors.

As key findings in this study, we demonstrated significant downregulation of tRNA-Val in liver tissue and plasma EVs from dogs with HCC versus both healthy controls and dogs with HCA. By contrast, we found no difference in tRNA-Val expression between dogs with HCA and healthy controls (in either tissue or plasma EVs). Our findings are consistent with reports that tRFs can act as diagnostic and prognostic biomarkers for human cancers, even at an early stage [11,12], and that this tRF has specific diagnostic utility for differentiating between benign and malignant canine MGTs [25]. Furthermore, various tRFs reportedly show diagnostic utility for a range of human cancers, interestingly including 5′-tiRNA-Glnm, which is significantly downregulated in HCC tissue versus tumor-adjacent tissue and in metastatic versus non-metastatic HCC [13]. In other examples, hsa-tsr 016141, tiRNA-Val-CAC-001, tRF-Glu-TTC-027, and tRF-5026a are preferentially downregulated in gastric cancer and inhibit the proliferation of this carcinoma [16,17,18,19]. tRF-Gly-CCC-046, tRF-Tyr-GTA-010, and tRF-Pro-TGG-001 are reportedly downregulated in breast cancer and have potential as diagnostic biomarkers for early-stage detection of this cancer [20].

To validate our findings in clinical samples, we investigated tRNA-Val expression in fast-proliferating (95-1044) and intermediate-proliferating (AZACH) canine HCC cell lines. tRNA-Val was preferentially decreased in 95-1044 and AZACH cells versus healthy control tissue, reflecting the trend in clinical tissue and normal liver samples. These findings are consistent with those for tRF expression in human cell lines: 3′U-tRF-Val-CAC promotes cell growth and migration in human ovarian cancer [14] and, as a novel tRF, tRF-Val is upregulated in gastric cancer tissues and cell lines, promoting cell proliferation and invasion and inhibiting cell apoptosis [15]. mt-tRFs are also reportedly significantly downregulated in canine oral melanoma tissues and cell lines versus controls [24], and tRNA-Val is downregulated in metastatic-site versus primary-site canine MGT cell lines [25].

Another noteworthy feature of this study was the particularly strong downregulation of tRNA-Val in plasma EVs from dogs with HCC versus healthy controls and dogs with HCA. EV-associated tRFs are evidently involved in cancer progression and have potential diagnostic utility to become biomarkers for various cancers [37,38,39]. EV cargos are important in intracellular communication, and EVs have been implicated in cancer cell communication proliferation and tumor and metastasis [26]. Our findings in plasma EVs, taken together with our overall expression findings in the fast-proliferating cell line, suggest that EVs play a similar role in canine HCC development and progression. Interestingly, plasma EV-associated tRNA-Ala-TGC-5-1 can distinguish canine oral melanoma patients from tumor-free dogs [30]. In human medicine, EV-associated tRFs are aberrantly expressed in non-small cell lung cancer, gastric carcinoma, and hypopharyngeal carcinoma [27,28,29]. Taken together with our findings, these reports indicate the utility of plasma EVs as a sample matrix for oncological molecular diagnostics.

Through bioinformatics analysis, we predicted genes that tRNA-Val may target, considering that tRFs have a gene-regulating ability similar to that of miRNAs [40]. The predicted target genes are involved in several biological processes. Downregulation of APLF (Aprataxin and PNK-like factor) inhibited cancer cell proliferation, altered cell cycle behavior, induced apoptosis, and impaired DNA repair ability [41]. CDC27, a significant player in the progression of HCC, is involved in malignancies through various mechanisms [42]. GTF2H1 (general transcription factor IIH subunit 1) is identified as a DNA repair-related gene that can predict liver cancer [43]. SMAD11, a member of the SMAD family, is a core protein in the TGF-β signaling cascade. The TGF-β/SMAD signaling pathway, where SMAD proteins like SMAD11 play a multifaceted role, is intricately involved in developing human HCC [44]. Dysregulation of ATXN1 (ataxin1) can lead to gene expression alteration that may contribute to tumorigenesis and cancer progression [45]. Studies have shown that changes in ATXN1 expression can affect cell growth processes critical to cancer development. MBNL1 (Muscleblind-like protein 1) has been implicated in cancer through its role in alternative splicing of genes involved in cell migration, DNA repair, and metastasis [46]. Upregulation of serine/arginine-rich splicing factor 2 (SRSF2) is significantly associated with a higher tumor grade and a poor prognosis in patients with HCC. In addition, SRSF2 increases the proliferation and tumorigenic potential of hepatoma cells by specifically controlling cancer-related splicing events [47]. UBA3 is crucial for the degradation of proteins involved in cell cycle control and apoptosis. Dysregulation of UBA3 can disrupt these processes, contributing to uncontrolled cell proliferation and survival in HCC [48]. Another study shows that UBA3 promotes the occurrence and development of intrahepatic cholangiocarcinoma through the MAPK signaling pathway [49]. tRNA-Val may be involved in the N-glycan biosynthesis and ubiquitin-mediated proteasome pathways, which play pivotal roles in protein trafficking and enzyme complex formation [50], and the regulation of many biological processes such as cell cycle control, proliferation, signal transduction, and transcription regulation [51,52], respectively. Recent research shows that N-glycan modification processes are evidently involved in epithelial–mesenchymal transition and extracellular matrix changes in liver cancer [53]. Another study shows that N-glycosylation patterns correlate with HCC subtypes [54]. Ubiquitination-proteasome-mediated degradation is a major pathway for regulating cellular proteins and is directly correlated with the severity of HCC [55].

Canines serve as excellent models for comparative oncology due to their spontaneous development of cancers similar to those found in humans. The histological types of these canine cancers closely resemble those seen in humans. Substantial evidence supports the notion that canines and humans share similar genes and pathways involved in the formation of tumors [56]. Although there isn’t direct evidence of canine tRNA-Val influencing human hepatocellular carcinoma (HCC), a broader understanding of tRNA dysregulation in cancer can provide valuable insights. Our findings on tRNA-Val expression could potentially contribute to studying human HCC.

This study has some limitations. We did not evaluate plasma tRF-Val as a biomarker, which means further investigations are necessary to fully elucidate the intrinsic mechanism of this tRF. The numbers of tissue and blood donors in this study (n = 28/20) were relatively small, and further investigation is needed in a larger study population.

## 5. Conclusions

In conclusion, we suggest that tRF-Val has potential diagnostic efficacy as a tRF biomarker for differentiating canine HCA from canine HCC. Our findings are of interest for research on new molecular diagnostic techniques and, ultimately, new therapeutic targets in canine and human oncology.

## Data Availability

All data from this study are included within this article and Supplementary Materials.

## Supplementary Materials

The following supporting information can be downloaded at: https://www.mdpi.com/article/10.3390/genes15081024/s1, Figure S1: ROC curves of tRNA-Val. ROC curve of the tRNA-Val for HCA (n = 5) vs. ctrl (n = 6). HCA; Hepatocellular adenoma, EVs; Extracellular vesicles; Table S1: HCC and HCA patient information; Table S2: Targeted genes of tRNA-Val with their seed sequence and binding locations; Table S3: Kyoto Encyclopedia Genes and Genomes (KEGG) pathway analysis of target genes of tRNA-Val; Table S4: Targeted genes of tRNA-Val with their seed sequence and binding locations.

## Abbreviations

Hepatocellular carcinoma: HCC; hepatocellular adenoma: HCA; tRNA-derived fragments: tRFs; noncoding RNAs: ncRNAs; mammary gland tumor: MGT; and extracellular vesicles: EVs.
