# Supplementary material for: Aberrantly Expressed tRNA-Val Fragments Can Distinguish Canine Hepatocellular Carcinoma from Canine Hepatocellular Adenoma
Source: Genes (Basel). 2024 Aug 4;15(8):1024. doi: 10.3390/genes15081024 (PMC11353709; doi:10.3390/genes15081024)
Supplement: Supplementary file 1 [file genes-15-01024-s001.zip › genes-3125872-supplementary.pdf]

Aberrantly expressed tRNA-Val fragments can distinguish canine hepatocellular carcinoma from canine hepatocellular adenoma

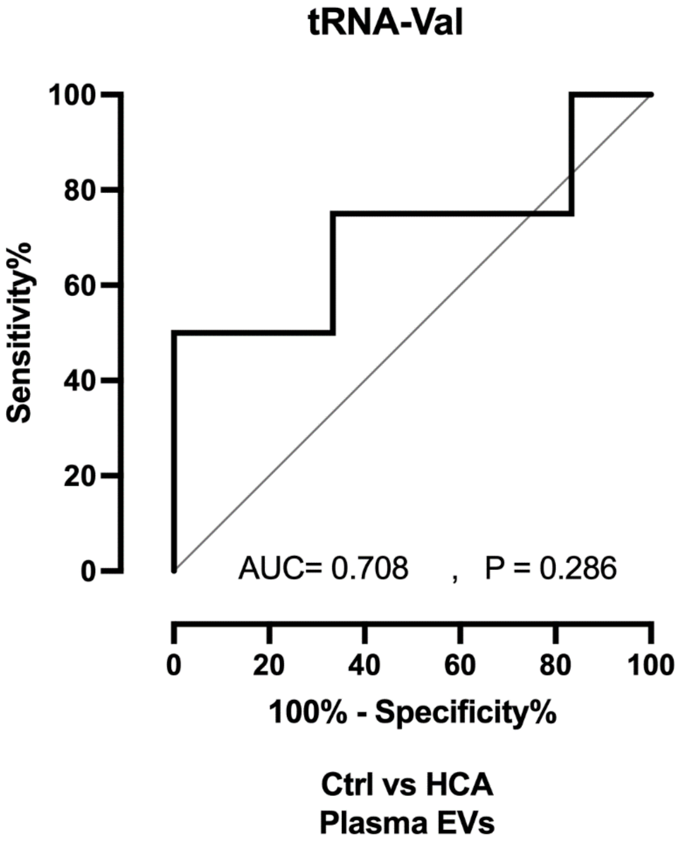

Figure S1. ROC curves of tRNA-Val. ROC curve of the tRNA-Val for HCA (n=5) vs. ctrl (n=6). Ctrl; control, HCA; Hepatocellular adenoma, EVs; Extracellular vesicles.

Supplementary Table S1. HCC and HCA patient information.

Number	Age	Disease	Sex	Castration/Spay	Breed	Tissue	Plasma
P1	11 Y 3 M	HCA	M	Yes	Mongrel		
P2	8 Y 1M	HCA	F	Yes	Miniature dachshund		
P3	10 Y 7 M	HCA	M	Yes	Toy poodle		
P4	12 Y 2 M	HCA	F	Yes	Shiba		
P5	11 Y 6 M	HCA	M	No	Miniature dachshund		
P6	12 Y 3 M	HCA	M	No	Miniature dachshund		
P7	11 Y 9 M	HCA	M	No	Mongrel		
P8	13 5 M	HCA	F	Yes	Miniature dachshund	15	5
P9	14 Y	HCA	M	No	Golden retriever		
P10	9 Y 2 M	HCA	F	Yes	Toy poodle		
P11	13 Y	HCA	F	Yes	Jack Russell terrier		
P12	11 Y 1 M	HCA	M	No	Miniature dachshund		
P13	12 Y 2 M	HCA	M	No	Mongrel		
P14	12 Y 3 M	HCA	M	No	Mongrel		
P15	10 Y 7 M	HCA	M	No	Shiba		
P16	12 Y 3 M	HCC	F	No	Chihuahua		
P17	11 Y 3 M	HCC	F	Yes	Miniature dachshund		
P18	14 Y	HCC	F	Yes	Mongrel		
P19	10 Y 8 M	HCC	M	Yes	Shiba		
P20	11 Y 7 M	HCC	M	Yes	Welsh corgi		
P21	10 Y 9 M	HCC	F	No	Mongrel		
P22	10 Y 3 M	HCC	F	No	Beagle		
P23	10 Y 9 M	HCC	F	No	Yorkshire terrier	13	9
P24	11 Y 6 M	HCC	M	No	Shiba		
P25	12 Y	HCC	F	No	Miniature schnauzer		
P26	11 Y 10 M	HCC	M	No	Yorkshire terrier		
P27	13 Y 10 M	HCC	F	No	Shetland sheepdog		
P28	11 Y 7 M	HCC	M	Yes	Mongrel		

Abbreviations[1]

[1] P; Patient, HCA; Hepatocellular adenoma, HCC; Hepatocellular carcinoma, F; Female; M; Male.

Supplementary Table S2: Gene ontology (GO) enrichment analysis of target genes of tRNA-Val.

GO				
**ID**	**Description**	**p-value**	**P-adjust**	**Gene ID**
GO:0000381	Regulation of alternative mRNA splicing, via spliceosome	0.00614944	0.379173043	SRSF2/MBNL1
GO:0000380	Alternative mRNA splicing, via spliceosome	0.00715982	0.379173043	SRSF2/MBNL1
GO:0048024	Regulation of mRNA splicing, via spliceosome	0.01233116	0.379173043	SRSF2/MBNL1
GO:0050684	Regulation of mRNA processing	0.01871341	0.379173043	SRSF2/MBNL1
GO:0030705	Cytoskeleton-dependent intracellular transport	0.0192516	0.379173043	HOOK1/KIF5C
GO:0007286	Spermatid development	0.02146922	0.379173043	HOOK1/STRBP
GO:0048515	Spermatid differentiation	0.02261632	0.379173043	HOOK1/STRBP
GO:0043484	Regulation of RNA splicing	0.02559299	0.379173043	SRSF2/MBNL1
GO:0045022	Early endosome to late endosome transport	0.03165494	0.379173043	HOOK1
GO:0048532	Anatomical structure arrangement	0.03165494	0.379173043	KCNA2
GO:0072525	Pyridine-containing compound biosynthetic process	0.03165494	0.379173043	PNPO
GO:0018196	Peptidyl-asparagine modification	0.03476699	0.379173043	MGAT2
GO:0018279	Protein n-linked glycosylation via asparagine	0.03476699	0.379173043	MGAT2
GO:0050432	Catecholamine secretion	0.03476699	0.379173043	KCNA2
GO:0050433	Regulation of catecholamine secretion	0.03476699	0.379173043	KCNA2
GO:0051443	Positive regulation of ubiquitin-protein transferase activity	0.03476699	0.379173043	DCUN1D1
GO:0006767	Water-soluble vitamin metabolic process	0.03786943	0.379173043	PNPO
GO:0015872	Dopamine transport	0.03786943	0.379173043	KCNA2
GO:0019228	Neuronal action potential	0.03786943	0.379173043	KCNA2
GO:0030521	Androgen receptor signaling pathway	0.03786943	0.379173043	RWDD1
GO:0098927	Vesicle-mediated transport between endosomal compartments	0.0409623	0.379173043	HOOK1
GO:0021602	Cranial nerve morphogenesis	0.04404563	0.379173043	KCNA2
GO:0030970	Retrograde protein transport, er to cytosol	0.04404563	0.379173043	FAF2
GO:0072595	Maintenance of protein localization in organelle	0.04404563	0.379173043	KDELR2
GO:1903513	Endoplasmic reticulum to cytosol transport	0.04404563	0.379173043	FAF2
GO:0007281	Germ cell development	0.0449163	0.379173043	HOOK1/STRBP
GO:1903311	Regulation of mRNA metabolic process	0.0449163	0.379173043	SRSF2/MBNL1
GO:0032355	Response to estradiol	0.04711944	0.379173043	SSTR1
GO:0043368	Positive t cell selection	0.04711944	0.379173043	THEMIS
GO:0051937	Catecholamine transport	0.04711944	0.379173043	KCNA2
GO:0072524	Pyridine-containing compound metabolic process	0.04711944	0.379173043	PNPO

Supplementary Table S3: Kyoto Encyclopedia Genes and Genomes (KEGG) pathway analysis of target genes of tRNA-Val.

KEGG				
**ID**	**Description**	**p-value**	**p-adjust**	**Gene ID**
cfa00513	Various types of N-glycan biosynthesis	0.002302947	0.058482864	MGAT2/MAN1C1
cfa00510	N-Glycan biosynthesis	0.003544416	0.058482864	MGAT2/MAN1C1
cfa04120	Ubiquitin mediated proteolysis	0.032010385	0.301550102	CDC27/UBA3
cfa00900	Terpenoid backbone biosynthesis	0.036551528	0.301550102	IDI1

Supplementary Table S4. Targeted genes of tRNA-Val with their seed sequence and binding locations.

Gene	Gene name	Seed location	Predicted target score
SAMD11	Sterile alpha motif domain containing 11	119	91
APLF	Aprataxin and PNKP like factor	40	83
SRSF2	Serine and arginine rich splicing factor 2	912	81
KDELR2	KDEL endoplasmic reticulum protein retention receptor 2	83	73
CDC27	Cell division cycle 27	2938	70
ARAP2	ArfGAP with RhoGAP domain, ankyrin repeat and PH domain 2	433, 1631	62
MBNL1	Muscle blind like splicing regulator 1	2880	54
GTF2H1	General transcription factor IIH subunit 1	628	53
UBA3	Ubiquitin-like modifier activating enzyme 3	32	53
ATXN1	Ataxin 1	2576	50
DCUN1D1	Defective in cullin neddylation 1 domain containing 1	48	50
